# Microbial signatures in follicular fluid and their association with fertilization success

**DOI:** 10.3389/frph.2026.1773092

**Published:** 2026-04-15

**Authors:** Gregor Weiss, Ekaterina Voroshilina, Manfred Koranda, Jennifer Blauensteiner, Michael Schenk

**Affiliations:** 1 Das Kinderwunsch Institut Schenk GmbH, Dobl, Austria; 2 Gottfried Schatz Research Center, Division of Cell Biology, Histology & Embryology, Medical University of Graz, Graz, Austria; 3 DNA-Technology AM, Yerevan, Armenia; 4 Department of Obstetrics and Gynecology, Medical University of Graz, Graz, Austria

**Keywords:** assisted reproduction, Femoflor, follicular fluid, Lactobacillus, microbiome

## Abstract

**Background:**

Emerging evidence suggests that the upper female reproductive tract is not sterile and that microbial signals within follicular fluid (FF) may influence oocyte competence. However, previous studies have largely relied on pooled FF samples or dominant follicles, limiting insight into follicle-specific associations with fertilization outcomes.

**Methods:**

In this exploratory paired study, follicular fluid samples were collected from 24 women undergoing IVF/ICSI treatment. For each patient, two FF samples were analyzed individually: one associated with a fertilized oocyte, and one associated with an oocyte that failed fertilization. Bacterial DNA and total bacterial load (TBL) were assessed using quantitative real-time PCR targeting predefined microbial taxa.

**Results:**

Bacterial DNA above the predefined detection threshold was identified in 39.6% of all FF samples. Notably, within this exploratory cohort, FF samples associated with fertilization failure were more frequently TBL-positive compared with FF samples linked to successful fertilization (70.8% vs. 8.3%). Follicles from the same patient often differed in bacterial DNA presence, indicating substantial intra-individual variability. Several bacterial taxa, including *Fannyhessea vaginae*, *Ureaplasma* spp., and *Lactobacillus* spp., were more frequently detected in FF samples associated with failed fertilization; however, no individual taxon showed a consistent association with outcome across all samples.

**Conclusion:**

In this paired follicle-level analysis, the absence of detectable bacterial DNA in follicular fluid was associated with fertilization outcome. These findings highlight follicle-level heterogeneity in microbial DNA detection and underscore the importance of follicle-specific analyses in reproductive microbiome research. Larger prospective studies are required to validate these observations and to clarify the biological mechanisms underlying follicular microbial signals.

## Graphical Abstract

**Figure:**
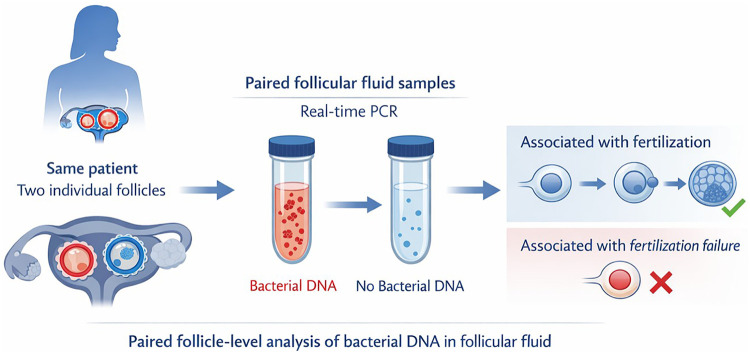
Schematic overview of the paired follicle-level study design and main findings. For each patient undergoing IVF/ICSI, two individual follicular fluid samples from separate follicles were analyzed using quantitative real-time PCR: one associated with a fertilized oocyte and one with a non-fertilized oocyte. Detection of bacterial DNA (total bacterial load, TBL) was more frequently observed in follicular fluid samples linked to fertilization failure, whereas samples without detectable bacterial DNA were predominantly associated with successful fertilization. These findings highlight intra-individual, follicle-specific heterogeneity in microbial DNA presence and its potential relevance for oocyte competence.

## Introduction

Infertility represents a growing global health concern, with an increasing number of couples experiencing difficulties in achieving pregnancy. Although assisted reproductive technologies (ART), including *in vitro* fertilization (IVF) and intracytoplasmic sperm injection (ICSI), have substantially improved treatment options, success rates remain limited. This has driven ongoing efforts to identify biological markers that reflect oocyte competence and early developmental potential beyond conventional morphological assessment (1). Among the components of the ovarian microenvironment, follicular fluid (FF) has emerged as a key biological matrix, as it directly surrounds the developing oocyte and integrates endocrine, metabolic, and cellular signals critical for folliculogenesis and fertilization (2–4).

Advances in molecular detection techniques have challenged the long-standing assumption that the upper female reproductive tract represents a sterile environment. Culture-independent methods, including next-generation sequencing and quantitative real-time PCR, have demonstrated the presence of microbial DNA throughout the reproductive tract, including the uterus, fallopian tubes, and ovaries, albeit at substantially lower abundance than in the vagina (5, 6). Within this context, Lactobacillus species have been widely associated with reproductive tract health, primarily through maintenance of an acidic microenvironment and inhibition of opportunistic pathogens (7, 8). However, deviations from Lactobacillus-dominant microbial profiles have been linked to dysbiosis and adverse reproductive outcomes, including endometrial inflammation, implantation failure, and pregnancy complications (9–11).

Follicular fluid has therefore gained increasing attention as a potential site of microbial influence on oocyte quality and ART outcomes. Several studies have reported the presence of bacterial DNA or viable microorganisms in FF samples collected during IVF procedures, with some suggesting associations between specific bacterial taxa and reduced fertilization rates or impaired embryo development (12–15). At the same time, other investigations have reported neutral or even favorable associations between certain microorganisms - particularly Lactobacillus species - and IVF outcomes, including higher embryo transfer or pregnancy rates (16, 17). A recent meta-analysis highlighted this heterogeneity, reporting only marginal differences in fertilization rates between FF-positive and FF-negative samples across studies (18). Together, these findings underscore the absence of a unified model describing how microbial signals within FF relate to oocyte competence and fertilization success.

A major limitation of existing studies is methodological heterogeneity, particularly regarding sample collection and analytical design. Many investigations rely on pooled FF samples, dominant follicles, or single samples per patient, approaches that obscure potential follicle-specific effects and intra-individual variability. Experimental evidence suggests that microorganisms or their metabolites may directly interfere with granulosa cell function and follicle-stimulating hormone (FSH) signaling, thereby affecting oocyte maturation at the level of individual follicles (16, 19). These observations raise the possibility that microbial influences on oocyte competence may be highly localized and not adequately captured by patient-level or pooled analyses. In addition, most microbiome studies in reproductive medicine depend on next-generation sequencing, a powerful but resource-intensive approach that is not readily applicable in routine clinical settings. In contrast, quantitative real-time PCR offers a standardized, sensitive, and cost-effective alternative for targeted microbial detection and may be more suitable for translational research and future clinical implementation.

The novelty of the present study lies in its paired, follicle-level design, enabling intra-individual comparison of fertilized and non-fertilized oocytes obtained from the same patient. By analyzing individually collected follicular fluid samples rather than pooled material or dominant follicles, this approach minimizes inter-individual confounding and allows exploration of localized microbial signals acting at the level of single follicles. While exploratory in nature, this design provides unique insight into intra-individual heterogeneity of follicular microbial DNA in the context of IVF/ICSI treatment and may help refine future hypothesis-driven and mechanistic studies.

## Material and methods

### Participants

A total of 24 women undergoing assisted reproductive treatment (ART) at the Kinderwunsch Institut Schenk GmbH in Dobl, Austria, participated in this study. Exclusion criteria included: [a] obesity (BMI > 30), [b] underweight with potential anorexia (BMI < 17.5), [c] endocrine conditions (e.g., polycystic ovary syndrome, diminished ovarian reserve, menopause, hypothalamic amenorrhea, congenital adrenal hyperplasia), [d] chronic inflammatory diseases, and [e] known genetic abnormalities. All participants gave written informed consent prior to participation. The study protocol and sample collection procedures were approved by the Ethics Committee of the Medical University of Graz (approval number: 34-186 ex21/22). Participants consented to the collection, storage, and retrospective scientific evaluation of their biological samples. Biosample collection was carried out according to the protocol established by Schenk et al. (20). In brief, controlled ovarian stimulation was initiated with follicle-stimulating hormone (FSH) on days 2–4 of the menstrual cycle, followed by GnRH antagonist treatment from days 5–6. Ovulation was triggered by an hCG injection administered 35 h prior to ultrasound-guided transvaginal follicular aspiration, performed between days 12–16 for follicles larger than 15 mm. Follicles were aspirated using a Steiner-Tan 17-gauge needle and flush system (IVFETFLEX.com HandelsgmbH & Co KG, Graz, Austria) under transvaginal ultrasound guidance (GE Healthcare Austria GmbH, Pfaffing, Austria). Retrieved follicular fluid (FF) was examined for oocytes in an IVF workstation (L24E, K-SYSTEMS Kivex Biotec A/S, Birkerød, Denmark) maintained at 37 °C and subsequently stored individually at −80 °C. Each follicular fluid sample corresponded to a single aspirated follicle and a single oocyte and was handled and stored individually to minimize the risk of cross-contamination. No pooling or mixing of follicular fluid samples was performed. From each participant, two follicular fluid samples were analyzed: one from a follicle that yielded a successfully fertilized oocyte, and another from a follicle where fertilization failed. In total, 48 samples were processed and analyzed. Baseline clinical characteristics and cycle parameters of the study cohort are summarized in Table 1.

**Table 1:** Baseline clinical characteristics and cycle parameters.

Variable	Value
Age (years)	32.6 ± 4.5
BMI (kg/m^2^)	21.4 ± 2.7
Infertility diagnosis	male factor (*n* = 11, 45.8%); unexplained (*n* = 11, 45.8%); PCOS (*n* = 2, 8.3%)
IVF/ICSI	IVF (*n* = 3, 12.5%), ICSI (*n* = 21, 87.5%)
Total gonadotropin dose (IU)	1,638 ± 483
Number of oocytes retrieved	10.4 ± 3.3

### Assessment of ART outcome

Embryo development was evaluated using the Gardner grading system. The blastocyst development rate (BDR), defined as the proportion of normally fertilized (2PN) zygotes reaching the blastocyst stage by day 5, was used to assess ART efficiency.

### DNA extraction

DNA was isolated using the PREP-NA-PLUS kit (DNA-Technology, Armenia). Prior to extraction, FF samples were deproteinized by centrifugation at 13,000 rpm for 10 min using a MiniSpin centrifuge (Eppendorf, Germany). The resulting pellet was resuspended in 100 µL of the kit's lysis buffer. A 50 µL aliquot was transferred to a clean tube containing 25 µL of additional lysis buffer, 5 µL of proteinase K (20 mg/mL, VWR Life Science, USA), and 120 µL of sterile 0.9% saline. Samples were incubated at 60 °C for 30 min, followed by heating at 95 °C for 10 min. After a brief centrifugation at 13,000 rpm for 1 min, 100 µL of supernatant was used for DNA extraction as per the kit instructions.

### Follicular fluid microbiota analysis

DNA from sexually transmitted obligate pathogens and opportunistic microorganisms (OM) present in follicular fluid (FF) samples was detected using two commercial real-time PCR assays: the Androflor® and Femoflor®16 kits (DNA-Technology, Armenia), run on the Dtprime 5M1 PCR system (DNA-Technology, Armenia). The Androflor® assay allows for the quantification of 24 bacterial taxa, while Femoflor®16 covers 16 (see Table 2). The bacterial load for each group was calculated automatically based on threshold cycle (Cq) values, expressed as a proportion of the total bacterial load (TBL). TBL represents cumulative bacterial DNA detected at the sample level, whereas taxon-specific signals depend on individual primer/probe targets and predefined detection limits. Sterile deionized water was used as a negative control (NC). Given the low microbial biomass of follicular fluid, particular attention was paid to minimizing false-positive PCR signals. Weak amplification signals above Cq 35 were occasionally observed in negative controls for selected bacterial groups, corresponding to <10^3^ genome equivalents per milliliter (GE/mL). To reduce the risk of environmental or reagent-derived background amplification, only samples with Cq values below 35 (≥10^3^ GE/mL) were classified as positive. Signals above this threshold were systematically interpreted as negative. This threshold was applied for sample-level classification only and does not imply biological absence of bacterial DNA below this level.

**Table 2:** Quantification of bacterial DNA in Androflor® and Femoflor®16.

Bacterium/group of bacteria	Androflor	Femoflor
*Lactobacillus spp.*	✓	✓
*Staphylococcus spp.*	✓	✓
*Streptococcus spp.*	✓	✓
*Corynebacterium spp.*	✓	✓ (in combination)
*Gardnerella vaginalis*	✓	✓ (in combination)
*Eubacterium spp.*	✓	✓
*Megasphaera spp./Veillonella spp.*	✓	✓ (in combination)
*Dialister spp.*	✓	✓ (in combination)
*Sneathia spp./Leptotrichia spp./Fusobacterium spp.*	✓	✓
*Ureaplasma urealyticum*	✓	✓ (as Ureaplasma spp.)
*Ureaplasma parvum*	✓	✓ (as Ureaplasma spp.)
*Mycoplasma hominis*	✓	✓
*Fannyhessea vaginae*	✓	✓
*Bacteroides spp./Porphyromonas spp./Prevotella spp.*	✓	✓ (in combination)
*Anaerococcus spp.*	✓	—
*Peptostreptococcus spp./Parvimonas spp.*	✓	✓
*Chlamydia trachomatis*	✓	—
*Trichomonas vaginalis*	✓	—
*Neisseria gonorrhoeae*	✓	—
*Mycoplasma genitalium*	✓	✓
*Enterobacteriaceae spp./Enterococcus spp.*	✓	✓
*Haemophilus spp.*	✓	—
*Pseudomonas aeruginosa/Ralstonia spp./Burkholderia spp.*	✓	—
*Mobiluncus spp.*	—	✓ (in combination)
*Lachnobacterium spp./Clostridium spp.*	—	✓
*Candida spp.*	✓	✓

An exception was made for *Ureaplasma urealyticum*, *Ureaplasma parvum*, and *Mycoplasma hominis*, for which no amplification occurred in the NC. Any amplification signal detected for these organisms was classified as a true positive.

While both PCR kits operate on similar principles, Femoflor®16 lacks primers for certain sexually transmitted pathogens and opportunistic bacteria that are targeted by Androflor®. Given the typically low bacterial load in FF samples, relying on a single test was not sufficient to ensure reliable detection. Thus, each sample was analyzed with both assays, and only those OMs consistently identified by both tests were regarded as confidently present.

### Statistical analysis

The comparison of TBL positive rates between samples with successful and unsuccessful fertilization was conducted by two-tailed Fisher's exact test. Further assessment of the classification performance of TBL in relation to fertilization outcome was performed by calculation of sensitivity, specificity, and overall accuracy. In addition to this, the agreement between the dichotomized measures of TBL and fertilization outcomes was assessed using Cohen's kappa statistics. For all statistical analyses, an alpha of 0.05 was used to evaluate statistically significant differences or associations. All analyses were performed using SPSS version 26 (IBM Inc., USA). Given the paired follicle-level design and limited sample size, statistical analyses were intended to be exploratory and descriptive rather than confirmatory. No multivariable adjustment for clinical covariates was performed due to the limited sample size and exploratory nature of the study.

## Results

Baseline demographic characteristics, infertility diagnoses, treatment modality (IVF vs. ICSI), and ovarian stimulation parameters are summarized in Table 1. TBL was detected in 19 of 48 samples (TBL-positive) with the range of positive results from 10^3.1^ to 10^4^ GE/mL. In most samples (29 of 48; 60.4%) we did not detect bacterial DNA in the quantity of more than 10^3^ GE/mL. These samples might have contained some traces of bacterial DNA; however, their quantity was less than 10^3^ GE/mL.

DNA of at least one specific bacterium or bacterial group was detected in 11 of 19 TBL-positive samples. Table 2 contains the information on the detection rate of determined groups of bacteria and TBL in FF samples. In the remaining eight TBL-positive samples, no taxon included in the Androflor® or Femoflor®16 panels exceeded the predefined detection threshold, despite total bacterial load being above the positivity cutoff.

From 1 to 4 bacterial groups were detected in TBL-positive FF samples simultaneously. Only one bacterium/group of bacteria was present in 6 samples: *Lactobacillus spp. –* in 2 samples, *F.vaginae –* in 2 samples; 2 more samples contained either *Streptococcus spp*. or *U.parvum.*

Two groups of bacteria were detected in 2 FF samples. One samples contained *Lactobacillus spp.*+*F.vaginae;* and the other *– Staphylococcus spp.*+*F.vaginae.*

Three groups of bacteria were detected in 2 FF samples: one sample contained *Lactobacillus spp.+ F.vaginae*+*U.parvum*; and the other – *G.vaginalis+F.vaginae*+*U.parvum.*

Four groups of bacteria (*Lactobacillus spp.+ G.vaginalis+Megasphaera spp./ Veilonella spp./ Dialister spp.+ Eubacterium spp.)* were detected in just one FF sample.

The quantity of *Lactobacillus spp., Staphylococcus spp., Streptococcus spp*., *G.vaginalis+Megasphaera spp./ Veilonella spp./ Dialister spp.+ Eubacterium spp.,* ranged from 10^3.1^ to 10^4^ GE/mL. In TBL-positive samples, individual bacterial taxa such as *Fannyhessea vaginae* and *Ureaplasma parvum* contributed at low relative abundance levels (10^1.3^–10^2.^⁸ GE/mL), despite total bacterial load exceeding the positivity threshold.

### Subgroup analyses of follicular fluid with/without fertilized oocytes

Next step was to find out if there was any difference in bacteria counts between the samples with or without fertilized oocytes. All the samples were divided into 2 groups: Group F + (*n* = 24) from fertilized oocytes and Group F- (*n* = 24) from non-fertilized oocytes.

**Figure 1:**
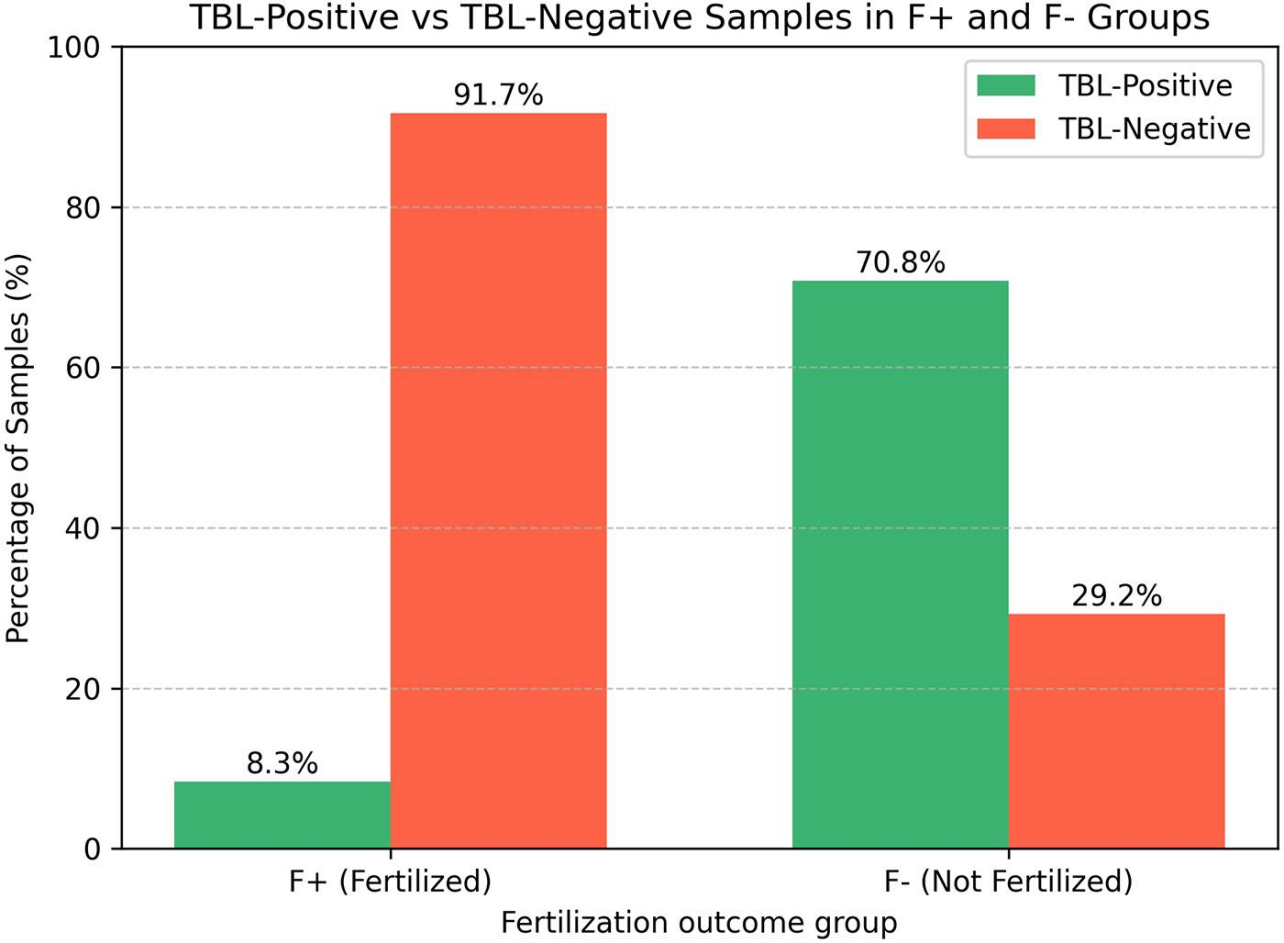
The TBL detection rate in follicular fluid samples from fertilized (Group F+) and non-fertilized (Group F-) oocytes, *p* < 0.001.

The most FF samples of Group F + (with oocyte fertilized) were TBL-negative: bacterial DNA was detected in only 2 (8.3%) samples (Figure 1). In both TBL-positive samples *Lactobacillus spp*. in quantity > 10^3^ GE/mL was found, while in one of them it was present in combination with *F.vaginae* and *U.parvum.*

In contrast, most FF samples of Group F- (from oocyte that failed fertilization) were TBL-positive: 17 (70.8%) of 24 (*p* < 0.001). At least one bacterium of bacteria group was found in 9 TBL-positive samples. The detection rate of individual bacteria is presented in Table 3. Only one of the following bacteria was detected in 5 FF samples if Group F-: *Lactobacillus spp*., *C.trachomatis*, *Streptococcus spp., F.vaginae* or *U.parvum.* The other 4 samples contained from 2 to 4 bacteria in various combinations: *Lactobacillus spp.+ F.vaginae; Staphylococcus spp.+ F.vaginae; G.vaginalis* *+* *F.vaginae* + *U.parvum; Lactobacillus spp.+ G.vaginalis+Megasphaera spp./ Veilonella spp./ Dialister spp.+ Eubacterium spp.*

**Table 3:** Detection rate of TBL and specific groups of bacteria in follicular fluid samples.

Bacterium/group of bacteria	Total, *n* = 48 *n* (%)	Group F+, *n* = 24 *n* (%)	Group F-, *n* = 24 *n* (%)
*Lactobacillus spp.*	5 (10.41%)	2 (8.33%)	3 (12.5%)
*Staphylococcus spp.*	1 (2.1%)	0	1 (4.2%)
*Streptococcus spp.*	1 (2.1%)	0	1 (4.2%)
*G. vaginalis*	2 (4.2%)	0	2 (8.3%)
*Eubacterium spp.*	1 (2.1%)	0	1 (4.2%)
*Megasphaera spp./ Veilonella spp./ Dialister spp.*	1 (2.1%)	0	1 (4.2%)
*U. parvum*	4 (8.3%)	1 (4.2%)	3 (12.5%)
*Atopobium cluster*	5 (10.4%)	1 (4.2%)	4 (16.7%)
*C. trachomatis*	1 (2.1%)	0	1 (4.2%)
TBL	19 (39.6%)	2 (8.3%)*	17 (70.8%)*

*p < 0.001.

### The influence of bacterial DNA presence on the fate of the oocyte

**Figure 2:**
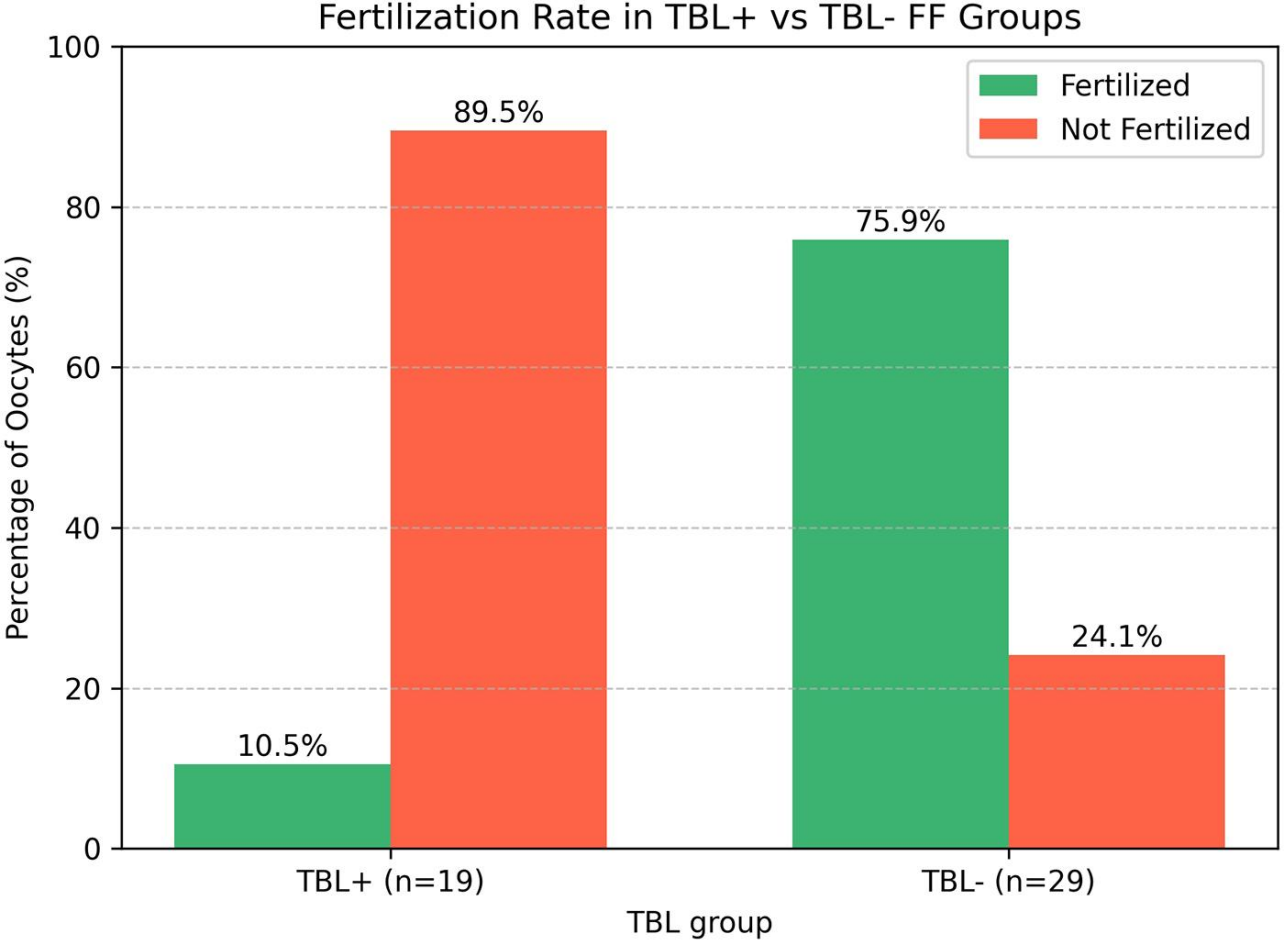
The fertilization rate of the oocytes collected from Group TBL-+ and Group TBL- follicular fluid samples.

All FF samples were divided into 2 groups depending on the presence of TBL in FF: Group TBL + (*n* = 19) and Group TBL- (*n* = 29). First, we evaluated the fertilization rate of the oocytes in these two groups (Figure 2).

Most oocytes with Group TBL + FF failed fertilization – 17 (89.5%) of 19. Only 2 (10.5%) oocytes were fertilized.

In contrast, the majority of oocytes with Group TBL- FF (Group 2) were fertilized – 22 (75.9%) of 29 Group 2 [*p* = 0.000 (1.7E-5)]; only 7 (24.1%) oocytes failed fertilization.

Then we analyzed the blastocyst development rate (BDR) in fertilized oocytes of Group TBL + and Group TBL-.

Both fertilized oocytes of Group TBL + developed into blastocysts, and despite their insufficient quality (3CC and 4CC according to Gardner scale), they were transferred as there were no other embryos available. Positive hCG was determined in one patient after transfer, but the no fetal heartbeat was detectable later. The other patient failed to demonstrate even biochemical pregnancy. Both fertilized oocytes in this group reached the blastocyst stage; however, given the very small number of observations, this finding is purely descriptive and does not allow any inference regarding blastocyst development rates or overall ART efficiency. 8 of 22 fertilized oocytes of Group TBL- developed into blastocysts of good and excellent quality, which were suitable for transfer; BDR was 27.6% for this group of samples. Biochemical pregnancy (confirmed by a positive serum hCG) was achieved in 3 of 8 patients who had embryo transfer; and a fetal heartbeat was determined in one patient.

### The follicular fluid microbiota analysis in paired samples and the success of reproductive medicine treatment

We analyzed the compatibility of microbiota composition in 24 pairs of FF; one sample in each pair was from fertilized oocyte, and the other from non-fertilized oocytes (Table 4).

**Table 4:** Outcome of ART depending on the presence of TBL in paired follicular fluid samples.

TBL status (paired samples)	Number of pairs	Transfer rate	hCG+	FHB+
Both TBL+	2	2	1	0
Both TBL-	7	3	0	0
TBL + TBL-	15	5	3	1

hCG, human chorionic gonadotropin; FHB, fetal heartbeat.

Paired samples from 7 patients were both TBL-negative. The quality of 4 blastocysts was insufficient, but the other 3 blastocysts were transferred. Unfortunately, the biochemical pregnancy was not confirmed by a positive serum hCG.

Paired samples from 2 patients were both TBL-positive, and we compared the spectrum of bacteria detected in FF from fertilized and non-fertilized oocytes of the same patient. In both cases they did not match each other. In one pair *Lactobacillus spp*. was detected in FF with fertilized oocyte, but no specific bacteria group was detected in non-fertilized FF. The embryo was transferred, but no positive serum hCG was obtained. In the other pair *Lactobacillus spp.+ F.vaginae*+*U.parvum* were detected in FF with fertilized oocyte, and *C.trachomatis* DNA was found in non-fertilized FF. The embryo was transferred, positive serum hCG was obtained, but no fetal heartbeat was determined.

Discrepant results were obtained in 15 paired samples: all non-fertilized FF were TBL-positive, while all FF with fertilized oocyte were TBL-negative. The quality of 10 blastocysts was insufficient, and the other 5 blastocysts were transferred. Biochemical pregnancy was confirmed by a positive serum hCG in 3 patients, and a fetal heartbeat was determined in one of them. These observations show that the absence of bacterial DNA in a particular follicle may be of greater importance for the success of ART in general.

### Association between absence of TBL and fertilization outcome

Among all investigated microbial parameters, only the TBL detection rate differed between the groups 1 and 2. The frequency of fertilization for oocytes from TBL-positive and TBL-negative FF samples was significantly different (10.5% vs. 75.8%, *p* < 0.001). We therefore explored the classification performance of TBL absence in distinguishing fertilized from non-fertilized oocytes in this dataset. For this reason, the calculation of sensitivity, specificity and overall accuracy was performed through the cross-tabulation procedure (Table 5). The TBL absence demonstrated sensitivity of 91.7% (true positive results), specificity of 70.8% (true negative results) and overall accuracy of 85.4% in predicting the successful fertilization. In addition to this calculation, we assessed the agreement between TBL absence and successful fertilization rates by Cohen's kappa. The kappa value was 0.625 (*p* < 0.001), which is considered as substantial agreement (21). These metrics describe classification performance within the present dataset and should not be interpreted as diagnostic validation or clinical prediction.

**Table 5:** Cross-tabulation of the TBL absence in follicular fluid samples and the successful fertilization and overall accuracy.

TBL absence in FF	Oocyte fertilization, *n* (%)		Total, *n*, (%)
	Yes	No	
Yes	22 (91.7%)	7 (29.2%)	29 (60.4%)
No	2 (8.3%)	17 (70.8%)	19 (39.6%)
Total	24 (100%)	24 (100%)	48 (100%)
$\mathrm{Overall}\;\mathrm{accuracy}\;=\frac{\left(TP+TN\right)}{\left(P+N\right)}*100=\frac{\left(22+17\right)}{\left(29+19\right)}*100=85.4\text{\%}$			

FF, follicular fluid, TP, true positive results; TN, true negative results; P, positive results; N, negative results.

Absence of TBL in FF samples according to real-time PCR results suggests the successful fertilization with 85.4% accuracy (Table 5).

## Discussion

In this exploratory paired study, we observed a markedly higher frequency of detectable bacterial DNA in FF samples associated with oocytes that failed fertilization compared with those associated with successful fertilization. Importantly, follicles retrieved from the same patient often differed in the presence or absence of bacterial DNA, supporting the concept that microbial signals within FF may act at the level of individual follicles rather than representing a uniform patient-level characteristic. Among the microbial parameters assessed, the absence of detectable TBL showed the strongest association with successful fertilization; however, no individual bacterial taxon consistently predicted outcome across all samples. The presence of TBL-positive follicular fluid samples without detectable taxon-specific signals highlights an important methodological consideration in targeted PCR-based microbiome analyses. Total bacterial load reflects cumulative bacterial DNA but does not provide taxonomic resolution. Such findings may indicate the presence of bacterial taxa not included in the detection panels, a diffuse low-level contribution of multiple organisms, or non-specific background amplification inherent to highly sensitive assays applied to low-biomass samples. Accordingly, TBL positivity should be interpreted as a global indicator of bacterial DNA presence rather than evidence for specific microbial colonization.

These findings contribute to an evolving and heterogeneous body of literature examining the follicular fluid microbiome in assisted reproduction. Previous studies have demonstrated that FF is not sterile and that microbial presence may be associated with altered fertilization rates and embryo development (14–16). At the same time, reports describing neutral or even favorable associations between certain microorganisms - particularly *Lactobacillus* species - and IVF outcomes highlight the complexity of microbial–reproductive interactions (16, 17). This heterogeneity is further reflected in meta-analytic data suggesting only marginal differences in fertilization rates between FF-positive and FF-negative samples (18). Our results are consistent with this mixed evidence and support the view that microbial effects within FF are context-dependent and may not be attributable to single taxa alone.

Experimental data provide potential biological plausibility for follicle-specific microbial influences. Beyond Lactobacillus species, the detection of non-Lactobacillus taxa and mixed microbial signals in follicular fluid may reflect localized inflammatory or metabolic disturbances within the follicular microenvironment. Experimental evidence suggests that bacterial components and metabolites can interfere with granulosa cell function, steroidogenesis, and follicle-stimulating hormone (FSH) receptor signaling, thereby impairing oocyte maturation and fertilization competence (16, 19). In addition, bacterial-derived inflammatory mediators may alter cytokine profiles within follicular fluid, potentially affecting oocyte–cumulus cell communication (22). Importantly, these effects are not necessarily species-specific but may arise from the cumulative microbial load or community composition, which could explain the absence of a consistent association with individual taxa in the present study.

The detection of Lactobacillus spp. in follicular fluid warrants cautious interpretation. While Lactobacillus dominance in the lower female reproductive tract is widely associated with reproductive tract health and microbial stability (7, 23, 24), genus-level detection in follicular fluid does not allow inference regarding functional relevance or biological effect. Different Lactobacillus species exhibit distinct metabolic and immunological properties, and PCR-based genus-level assays cannot distinguish between potentially beneficial, neutral, or incidental species. Moreover, detection of Lactobacillus DNA in follicular fluid may reflect procedural contamination routes during transvaginal aspiration rather than true follicular colonization, particularly in low-biomass samples. Accordingly, Lactobacillus spp. detection in the present study should not be interpreted as inherently beneficial or detrimental to fertilization outcome.

To establish causality between follicular microbial signals and oocyte competence, future studies should incorporate functional experimental approaches. For example, controlled *in vitro* exposure of oocytes or granulosa cell models to follicular fluid with defined microbial profiles could help disentangle direct microbial effects from indirect inflammatory or hormonal mechanisms. Such experiments, combined with longitudinal clinical studies, will be critical to translate observational findings into mechanistic understanding.

Several limitations must be acknowledged. First, the sample size was modest, and the study was not powered to detect associations for individual bacterial taxa. The exploratory paired design was chosen to minimize inter-individual confounding rather than to support population-level inference or diagnostic conclusions.

Second, the present study is subject to limitations inherent to low-biomass microbiome research. Although strict sterile clinical and laboratory procedures were applied and conservative detection thresholds were used, procedural contamination cannot be entirely excluded. The absence of extraction blank controls and aspiration flush controls represents an important limitation and should be addressed in future studies specifically designed for follicular fluid microbiome analysis. Importantly, the frequent observation of discordant microbial DNA detection between paired follicles obtained from the same patient argues against uniform procedural contamination and supports the relevance of follicle-specific microbial signals.

Third, the targeted nature of real-time PCR restricts detection to predefined bacterial taxa and does not capture the full microbial diversity present in follicular fluid. In addition, the dichotomization of bacterial DNA detection using a predefined Cq threshold represents a methodological simplification and may overlook biologically relevant low-level signals. Threshold robustness and quantitative interpretation across a broader dynamic range should therefore be addressed in future studies with larger sample sizes and comprehensive contamination controls.

Fourth, all follicular fluid samples were obtained under conditions of controlled ovarian stimulation. Gonadotropin exposure may influence follicular physiology, immune signaling, and microbial DNA detection within the follicular environment. However, follicular aspiration from unstimulated cycles is not ethically or practically feasible in routine clinical settings without medical indication. Future studies using appropriate experimental models or alternative sampling strategies will be required to clarify the impact of hormonal stimulation on follicular microbial signals.

Finally, several clinically relevant factors that may influence fertilization outcomes were not adjusted for in the present analysis. Due to the exploratory design and limited sample size, stratification by infertility diagnosis, treatment modality, semen parameters, antibiotic exposure, follicle size at aspiration, or blood contamination of follicular fluid was not feasible. These variables may act as confounders and should be systematically addressed in larger, prospective studies using paired or mixed-effects statistical models.

Despite these limitations, this study highlights the importance of follicle-specific analyses in reproductive microbiome research and demonstrates the feasibility of using quantitative real-time PCR to explore microbial signals in FF. Larger, prospective studies integrating species-level resolution and functional readouts will be required to validate these findings and to clarify the mechanisms by which microbial signals may influence oocyte competence and fertilization outcomes.

## Data Availability

The raw data supporting the conclusions of this article will be made available by the authors, without undue reservation.

## References

[1] Inhorn MC Patrizio P. Infertility around the globe: new thinking on gender, reproductive technologies and global movements in the 21st century. Hum Reprod Update. (2015) 21(4):411–26. 10.1093/humupd/dmv016 25801630

[2] Clarke HG Hope SA Byers S Rodgers RJ. Formation of ovarian follicular fluid may be due to the osmotic potential of large glycosaminoglycans and proteoglycans. Reproduction. (2006) 132(1):119–31. 10.1530/rep.1.00960 16816338

[3] Dumesic DA Meldrum DR Katz-Jaffe MG Krisher RL Schoolcraft WB. Oocyte environment: follicular fluid and cumulus cells are critical for oocyte health. Fertil Steril. (2015) 103(2):303–16. 10.1016/j.fertnstert.2014.11.015 25497448

[4] Revelli A Piane LD Casano S Molinari E Massobrio M Rinaudo P. Follicular fluid content and oocyte quality: from single biochemical markers to metabolomics. Reprod Biol Endocrinol. (2009) 7:40. 10.1186/1477-7827-7-40 19413899 PMC2685803

[5] Chen C Song X Wei W Zhong H Dai J Lan Z The microbiota continuum along the female reproductive tract and its relation to uterine-related diseases. Nat Commun. (2017) 8(1):875. 10.1038/s41467-017-00901-0 29042534 PMC5645390

[6] Moreno I Simon C. Deciphering the effect of reproductive tract microbiota on human reproduction. Reprod Med Biol. (2018) 18(1):40–50. 10.1002/rmb2.12249 30655720 PMC6332752

[7] Linhares IM Summers PR Larsen B Giraldo PC Witkin SS. Contemporary perspectives on vaginal pH and lactobacilli. Am J Obstet Gynecol. (2011) 204(2):120.e1–5. 10.1016/j.ajog.2010.07.010 20832044

[8] Miles SM Hardy BL Merrell DS. Investigation of the microbiota of the reproductive tract in women undergoing a total hysterectomy and bilateral salpingo-oopherectomy. Fertil Steril. (2017) 107(3):813–820.e1. 10.1016/j.fertnstert.2016.11.028 28069180

[9] Hashimoto T Kyono K. Does dysbiotic endometrium affect blastocyst implantation in IVF patients? J Assist Reprod Genet. (2019) 36(12):2471–9. 10.1007/s10815-019-01630-7 31741256 PMC6910901

[10] Moreno I Codoñer FM Vilella F Valbuena D Martinez-Blanch JF Jimenez-Almazán J Evidence that the endometrial microbiota has an effect on implantation success or failure. Am J Obstet Gynecol. (2016) 215(6):684–703. 10.1016/j.ajog.2016.09.075 27717732

[11] Moreno I Franasiak JM. Endometrial microbiota-new player in town. Fertil Steril. (2017) 108(1):32–9. 10.1016/j.fertnstert.2017.05.034 28602480

[12] Kabodmehri R Sharami SH Tavakoli S Donyaei-Mobarrez Y Zahiri Sorouri Z Ghanami Gashti N Association between follicular fluid bacteria with inflammatory markers of the complete blood count and the outcomes of assisted reproductive technology in women with endometriosis: a case–control study. Health Sci Rep. (2024) 7(2):e1874. 10.1002/hsr2.1874 38343663 PMC10853489

[13] Pelzer ES Allan JA Waterhouse MA Ross T Beagley KW Knox CL. Microorganisms within human follicular fluid: effects on IVF. PLoS One. (2013) 8(3):e59062. 10.1371/journal.pone.0059062 23554970 PMC3595219

[14] Pelzer ES Allan JA Cunningham K Mengersen K Allan JM Launchbury T Microbial colonization of follicular fluid: alterations in cytokine expression and adverse assisted reproduction technology outcomes. Hum Reprod. (2011) 26(7):1799–812. 10.1093/humrep/der108 21511711

[15] Wei W Zhou Y Zuo H Li M Pan Z Liu B Characterization of the follicular fluid microbiota based on culturomics and sequencing analysis. J Med Microbiol. (2023) 72(8):001741. 10.1099/jmm.0.001741 37578331

[16] Pelzer ES Harris JE Allan JA Waterhouse MA Ross T Beagley KW TUNEL analysis of DNA fragmentation in mouse unfertilized oocytes: the effect of microorganisms within human follicular fluid collected during IVF cycles. J Reprod Immunol. (2013) 99(1–2):69–79. 10.1016/j.jri.2013.07.004 23972717

[17] Wu YR Dong YH Liu CJ Tang XD Zhang NN Shen J Microbiological composition of follicular fluid in patients undergoing IVF and its association with infertility. Am J Reprod Immunol. (2023) 89(3):e13652. 10.1111/aji.13652 36397134

[18] Ou S Liao M Cui L Du Y Zhao L Peng C Associations between microbial presence in follicular fluid with IVF outcomes: a systematic review and meta-analysis. J Assist Reprod Genet. (2023) 40(11):2501–11. 10.1007/s10815-023-02912-x 37688752 PMC10643413

[19] Sluss PM Reichert LE. Presence of bacteria in porcine follicular fluid and their ability to generate an inhibitor of follicle-stimulating hormone binding to receptor. Biol Reprod. (1983) 29(2):335–41. 10.1095/biolreprod29.2.335 6315092

[20] Schenk M Huppertz B Obermayer-Pietsch B Kastelic D Hörmann-Kröpfl M Weiss G. Biobanking of different body fluids within the frame of IVF-a standard operating procedure to improve reproductive biology research. J Assist Reprod Genet. (2017) 34(2):283–90. 10.1007/s10815-016-0847-5 27889868 PMC5306411

[21] Landis JR Koch GG. The measurement of observer agreement for categorical data. Biometrics. (1977) 33(1):159–74. 10.2307/2529310 843571

[22] Tariq A Seekford ZK Bromfield JJ. Inflammation during oocyte maturation reduces developmental competence and increases apoptosis in blastocysts. Biol Reprod. (2025) 112(3):420–33. 10.1093/biolre/ioae180 39665379

[23] Ng BK Chuah JN Cheah FC Ismail M Tan NA Wong GC Maternal and fetal outcomes of pregnant women with bacterial vaginosis. Front Surg. (2023) 10:1084867. 10.3389/fsurg.2023.1084867 36860946 PMC9968788

[24] Verstraelen H Verhelst R Claeys G De Backer E Temmerman M Vaneechoutte M. Longitudinal analysis of the vaginal microflora in pregnancy suggests that L. crispatus promotes the stability of the normal vaginal microflora and that L. gasseri and/or L. iners are more conducive to the occurrence of abnormal vaginal microflora. BMC Microbiol. (2009) 9:116. 10.1186/1471-2180-9-116 19490622 PMC2698831

